# Norepinephrine may promote the progression of *Fusobacterium nucleatum* related colorectal cancer via quorum sensing signalling

**DOI:** 10.1080/21505594.2024.2350904

**Published:** 2024-05-09

**Authors:** Xinhao Du, Zhenzhen Tang, Li Yan, Ling Zhang, Qiao Zheng, Xianghao Zeng, Qing Hu, Qian Tian, Lanfan Liang, Xinyu Zhao, Jun Li, Ming Zhao, Xiangsheng Fu

**Affiliations:** a Department of Gastroenterology, Clinical Medical College and the First Affiliated Hospital of Chengdu Medical College, Chengdu, Sichuan, P.R. China; b Clinical Medical College, North Sichuan Medical College, Nanchong, Sichuan, P.R. China

**Keywords:** Stress, norepinephrine, *Fusobacterium nucleatum*, colorectal cancer, autoinducer-2

## Abstract

*Fusobacterium nucleatum (F. nucleatum)* is closely correlated with tumorigenesis in colorectal cancer (CRC). We aimed to investigate the effects of host norepinephrine on the carcinogenicity of *F. nucleatum* in CRC and reveal the underlying mechanism. The results revealed that both norepinephrine and bacterial quorum sensing (QS) molecule auto-inducer-2 (AI-2) were positively associated with the progression of *F. nucleatum* related CRC (*p* < 0.01). *In vitro* studies, norepinephrine induced upregulation of QS-associated genes and promoted the virulence and proliferation of *F. nucleatum*. Moreover, chronic stress significantly increased the colon tumour burden of Apc^Min/+^ mice infected with *F. nucleatum* (*p* < 0.01), which was decreased by a catecholamine inhibitor (*p* < 0.001). Our findings suggest that stress-induced norepinephrine may promote the progression of *F. nucleatum* related CRC via bacterial QS signalling. These preliminary data provide a novel strategy for the management of pathogenic bacteria by targeting host hormones-bacterial QS inter-kingdom signalling.

## Introduction

It has been estimated that over 100 trillion microbes reside in the human intestine as symbionts, and that they are mutually beneficial to the host [1]. However, dysregulation of the gut microbiota is related to a variety of diseases, including colorectal cancer (CRC), which is the third leading cause of cancer-related deaths worldwide [2]. An increasing body of evidence has demonstrated that *F. nucleatum*, previously recognized as a major constituent of the normal oral microbial community, is enriched in tumour tissues and stools of CRC patients compared to healthy controls [3–6]. An ectopic accumulation of *F. nucleatum* leads to a proinflammatory microenvironment that promotes CRC cellular proliferation [6,7]. *F. nucleatum* adhere to and invade epithelial cells via *fadA* and promotes CRC by modulating E-cadherin/β-catenin signalling [8–10]. Besides, *F. nucleatum* is capable of stimulating LC3-II expression, autophagic flux, and autophagosome synthesis in CRC cells which results in chemoresistance [11]. As an opportunistic pathogen, *F. nucleatum* also exists in healthy gut [12]. However, the mechanism that determines the pathogenicity of *F. nucleatum* are not entirely clear.

Interbacterial communication relies on the quorum sensing (QS) system, which utilizes signal molecules termed autoinducers (AIs) to regulate bacterial behaviour such as growth, virulence, and pathogenicity [13,14]. As a major molecule of QS, autoinducer-2 (AI-2), a furanosyl borate diester or tetrahydroxy furan [15], has been reported to be recognized by many Gram-negative and Gram-positive bacteria [16] and is closely correlated with the pathogenicity of a variety of bacteria in the gastrointestinal tract [17]. A previous study showed that *F. nucleatum* derived AI-2 mediate the interaction between the *F. nucleatum* and periodontopathogens via biofilm formation, coaggregation and enhanced expression of adhesion molecules [18]. AI-2 also regulating macrophage infiltration into tumour microenvironments and induces CRC occurrence [19]. Our previous work showed that bacteria-derived AI-2 was associated with tumour immunity in CRC patients through inducing tumour-associated macrophages and reducing CD4/CD8 ratio in a TNFSF9-dependent manner [20]. Recently, we found that gut microbiota-derived AI-2 could modulate lung inflammation through the gut-lung axis [21]. Nevertheless, few studies have addressed the influence of host factors on bacterial QS [22], which remains an important area of investigation in a variety of diseases caused by microbiota imbalance, such as CRC.

It has been well recognized that a variety of hormones are secreted into the gastrointestinal tract, and influence the gastroenteric function [23,24]. Chronic stress induces norepinephrine (NE) secretion from the enteric nervous system via the brain-gut axis [25]. NE is partly produced in the enteric nervous system [26] and reaches concentrations up to 50 ng/g in the colon [27]. Gastrointestinal stress hormones can not only modulate intestinal motility, endocrine signalling, and immunity but also regulate the composition and behaviour of gut microbiota [28]. It has been reported that NE can promote growth, virulence, and QS signalling in some anaerobic bacteria [29–31]. Thus, we hypothesized that host NE might promote the progression of *F. nucleatum* related CRC involving bacterial QS system.

In the current study, we examined the abundance of *F. nucleatum* and concentrations of AI-2 and NE in 40 CRC patients at different stages. *In vitro* studies were used to investigate the role of NE in the pathogenicity of *F. nucleatum*, which was further explored in Apc^Min/+^ mouse model fed with *F. nucleatum*. Our results suggest that NE promotes the progression of *F. nucleatum* related CRC, possibly through bacterial QS signalling.

## Materials and methods

### Clinical sample collection

Tumors and adjacent normal tissues were collected from 40 patients with CRC who underwent surgery at the Affiliated Hospital of North Sichuan Medical College (Nanchong, China). Fecal samples were collected from CRC patients before surgery and from 40 healthy volunteers. All samples were processed as previously described [32]. Clinicopathological characteristics of the samples are listed in Supplementary Table S1. CRC patients and healthy controls were age- and sex-matched. Informed consent was obtained from all participants, and the study was approved by the Institutional Review Board of the Affiliated Hospital of North Sichuan Medical College (Nanchong, China) (Approval ID: No. K2017027).

### Quantification of F. nucleatum by qRT-PCR

qRT-PCR was used to calculate the relative abundance of *F. nucleatum* in tissues [33,34]. Total DNA was extracted from the tissues using the kit (DP304–03, TIANGEN, China) according to the manufacturer’s instructions and was used as the template for qRT-PCR. qRT-PCR using specific primers targeting *F. nucleatum* was performed to estimate the relative abundance of *F. nucleatum*. *Gapdh* was set as a reference gene to normalize the *F. nucleatum* abundance. The relative abundance of *F. nucleatum* was calculated using the 2^−ΔΔCT^ method [35]. Each experiment was repeated in triplicate.

Primers were listed as follows:

***F. nucleatum***:forward: 5’-ACCTAAGGGAGAAACAGAACCA-3;’

***F. nucleatum***:reverse: 5’-CCTGCCTTTAATTCATCTCCAT-3.’

***Gapdh***:forward: 5’-ACGGGAAGCTCACTGGCATGGCCTT-3;’

***Gapdh***:reverse:5’-CATGAGGTCCACCACCCTGTTGCTG-3.’

### Analysis of Fusobacterium adhesin A (fadA) and luxS expression by qRT-PCR

The bacterial cultures with OD 600 = 0.7, were harvested for RNA extraction using TRIzol (R0016; Beyotime Biotechnology, China) according to the manufacturer’s protocol [5]. qRT-PCR using specific primers targeting *luxS* or *fadA* were performed to estimate the level of *luxS* or *fadA*. Primers targeting *F. nucleatum*-specific 16S rRNA was set as a reference to normalize the the level of *fadA*. The relative level of *luxS* or *fadA* was calculated using the 2^−ΔΔCT^ method [35] and normalized to the *F. nucleatum*-specific 16S rRNA. To quantify the *fadA* or *luxS* gene copies, plasmid carrying *fadA* or *luxS* was serially diluted to 10^2^–10^8^ *fadA* or *luxS* copies/ml and used to generate standard curves for Ct values. The *fadA* or *luxS* gene copies in the clinical samples were calculated based on the standard curves. Each experiment was repeated in triplicate [9].

Primers were listed as follows:

***FadA***:forward: 5’-GAAGAAAGAGCACAAGCTGA-3;’

***FadA***:reverse:5’- GCTTGAAGTCTTTGAGCTCT-3.’

***LuxS***:forward: 5’-AAGCCCCTTATGTGCGTATC-3;’

***LuxS***:reverse: 5’ -GGATAATCTCAGCGACTAAATGC-3;’

**16s rRNA**:forward: 5’-ACCTAAGGGAGAAACAGAACCA-3;’

**16s rRNA**:reverse: 5’-CCTGCCTTTAATTCATCTCCAT-3.’

### Fluorescent in situ hybridization (FISH)

FISH was performed as previously described [36]. The sequence of the FITC – labelled *F. nucleatum* - targeted probe, FUS664, was: 5ʹ - CGCAATACAGAGTTGAGCCCTGC − 3ʹ. To quantify *F. nucleatum* abundance, three blind observers assessed five random fields with 200 × magnification per sample and calculated the density of *F. nucleatum*.

### Bacteria culture and drug treatment

The *F. nucleatum* used in our study was separated anaerobically from a right-sided colon tumour and a single isolate *F. nucleatum* (F01) was obtained in our previous study [5]. The F01 strain was sequenced and submitted to GenBank (accession number: SUB1766768 Seq01 KX692281). The cells were cultured in fastidious anaerobic medium (FAB) supplemented with 10% foetal bovine serum in an incubator (containing 85% N_2_, 10% H_2_ and 5% CO_2_, 37°C) (Yuejin, Shanghai, China). *Vibrio harveyi* (*V. harveyi*) BB170 was obtained from Guangdong Culture Collection Center (Guangzhou, China) and cultured at 30°C in autoinducer bioassay (AB) medium. NE and propranolol were obtained from Solarbio, China, and used to treat *F. nucleatum* at 1 ng/ml.

### Bacterial growth assays

A serial dilution series of *F. nucleatum* (10^−5^-10^−8^) in 1 ml medium was transferred to agar plates containing 10% defibrillated sheep blood. After incubation at 37°C for 48 h in an anaerobic chamber, bacterial colonies were counted manually.

### AI-2 determination

*V. harveyi* strain BB170 was used to detect the concentration of AI-2, as previously described [37,38]. A standard solution of dihydroxy-2, 3-pentanedione (DPD) (710374–30–4, Omm Scientific Inc., USA), an AI-2 precursor, was prepared as previously described [39]. Briefly, 10 µl of each sample was added to 90 µl of *V. harveyi* BB170 followed by incubation at 30°C for 4.5 hours in a shaker at 160 rpm. The concentration of AI-2 was calculated based on the bioluminescence value.

### Mouse CRC models

All animal experiments and protocols were approved by the Animal Care and Animal Experiment Ethics Committee of the North Sichuan Medical College (Approval ID: No. 201702004). Six-week-old male C57BL/6-Apc^Min/+^ mice were purchased from Jiangsu JiCui Yaokang Biotechnology Co., Ltd. and raised in an SPF environment at the Animal Experiment Center of North Sichuan Medical College. Fifty C57BL/6-Apc^Min/+^ mice were randomly assigned into five groups: (1) Control; (2) *F. nucleatum*; (3) *F. nucleatum* + D-Ribose (D-Rib); (4) *F. nucleatum* + Stress; (5) *F. nucleatum* + Stress + α-methyl-p-tyrosine (AMPT). The C57BL/6-ApcMin/+ mice were fed by gavage with *F. nucleatum* at approximately 109 colony-forming units (cfu) of *F. nucleatum* in FAB medium daily for 8 consecutive weeks. D-Rib, an AI-2 inhibitor by competitively binding with the AI-2 receptor RbsB [40], was diluted in drinking water (100 mM final concentration) and replaced every other day. On Day 40 of *F. nucleatum* administration, mice were subjected to a chronic stress paradigm. For the chronic stress model, mice administered *F. nucleatum* for 40 days daily were placed in a well-ventilated 50 mL centrifuge tube for 2 h per day for 20 consecutive days [41]. Contemporaneously, AMPT (OKA, China, 100 mg/kg) was administered by intraperitoneal injection for 20 consecutive days.

### Enzyme-linked immunosorbent assay (ELISA)

ELISA was used to detect the concentration of NE in human and mouse tumours [42,43]. Intestinal tumour tissues were mixed with PBS at a ratio of 1:9 (w/v). After homogenization and centrifugation (4°C, 5000 rpm, 20 min), the supernatant was collected and stored at −20°C. To determine the concentration of NE, 20 μl supernatant was subjected to mouse or human NE ELISA kit (Andygen, U.S. AD11667Hu or AD3383Mo). A standard curve was generated using the reference standard set provided by the kit. The samples were measured according to the manufacturer’s instructions accompanying the kit. The NE concentration was calculated using a standard curve.

### Transcriptome sequencing

The total RNA was extracted from *F. nucleatum* with/without NE treatment. The mRNA was purified from total RNA for library preparation. The mRNA was randomly fragmented and used as the template for the synthesis of first strand cDNA. The second strand cDNA was synthesized with dNTP that containing dUTP instead of dTTP in the DNA polymerase I system. After adenylation and adaptor ligation, the second strand cDNA was fragmented, followed by purification with AMPure XP system (Beckman Coulter, Beverly, USA) to obtain fragments of 370 ~ 420 bp in length. Then these fragments were amplified by PCR and purified for quality control on the Agilent Bioanalyzer 2100. Next, the qualified index-coded samples were clustered using TruSeq PE Cluster Kit v3-cBot-HS (Illumina) according to the provided protocol. At last, the library sequencing was performed on an Illumina Novaseq platform and the paired-end reads of 150 bp were generated. For the transcriptome sequencing analysis, the data were blasted with the reference sequence (NZ_CP053468.1). The gene transcription level was evaluated with the RSEM package, and the up- or down-regulated genes were selected for functional analysis.

## Results

### F. nucleatum and AI-2 accumulated with the progression of CRC

**Figure 1.**
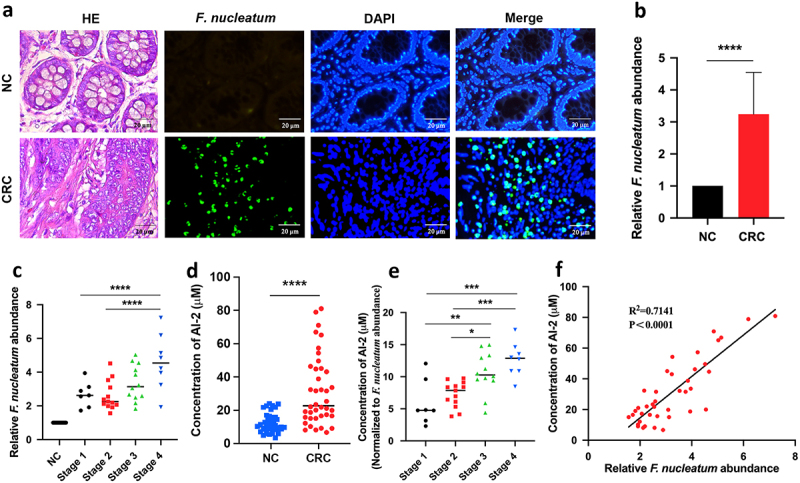
*F*. *nucleatum* increased along with CRC progression and AI-2 accumulation (a) Representative images of FISH detecting *F. nucleatum* in CRC and NC tissues. (b) qRT-PCR data showed the significantly increased abundance of *F. nucleatum* in CRC tissues (*n* = 40) compared with that in NC tissues (*n* = 40). The relative abundance of *F. nucleatum* in tissues was estimated by qRT-PCR using the specific primers targeting *F. nucleatum*, and primers targeting *gapdh* were used to confirm the equal quantity of tissues. (c) qRT-PCR data showed the increased abundance of *F. nucleatum* along with the cancer progression. (d) the concentration of AI-2 is significantly higher in CRC tissues compared with NC tissues, and (e) increased along with the cancer progression. (f) Pearson’s correlation analysis showed a positive correlation between *F. nucleatum* abundance and AI-2 concentration in tissues of CRC patients.

We used Fluorescent in Situ Hybridization (FISH) analysis to label *F. nucleatum* in the tumours and adjacent normal tissues of patients with CRC. Consistent with previous findings, we observed an accumulation of *F. nucleatum* in tumour tissue and less accumulation in nearby non-neoplastic tissues (Figure 1a). To quantify the abundance of *F. nucleatum* in each sample, qRT-PCR analysis was performed, and the results were consistent with the FISH data (*p* < 0.0001) (Figure 1b). Furthermore, we noted that the abundance of *F. nucleatum* was higher in advanced stage 4 tumours than in the early clinical stages (stages 1 and 2, *p* < 0.01) (Figure 1c), suggesting that *F. nucleatum* may be correlated with CRC progression.

****p < 0.0001, **p < 0.01. *p < 0.05. *F. nucleatum*, *Fusobacterium nucleatum*; CRC, colorectal cancer; AI-2, auto-inducer-2; FISH, Fluorescent in Situ Hybridization; NC, adjacent normal control.

Next, we evaluated the concentration of AI-2, a major molecule in the bacterial QS system. Concomitant with increased *F. nucleatum* abundance in tumour tissue, the concentration of AI-2 in tumour tissue was significantly higher than that in adjacent normal tissue (*p* < 0.0001) (Figure 1d) and continued to increase along with the clinical stage of cancer (Figure 1e). In addition, AI-2 concentration was significantly higher in stools from CRC patients (*n* = 40) than in healthy volunteers (*n* = 40, *p* < 0.0001) (Fig. S1A) and increased with CRC progression (Fig. S1B). Notably, Pearson’s correlation analysis showed a positive correlation between *F. nucleatum* abundance and AI-2 concentration in CRC tumour samples (*p* < 0.0001) (Figure 1f).

Taken together, these results suggest that the bacterial QS molecule AI-2 May be associated with the progression of *F. nucleatum* related CRC.

### NE increased with the progression of *F. nucleatum* related CRC

**Figure 2.**
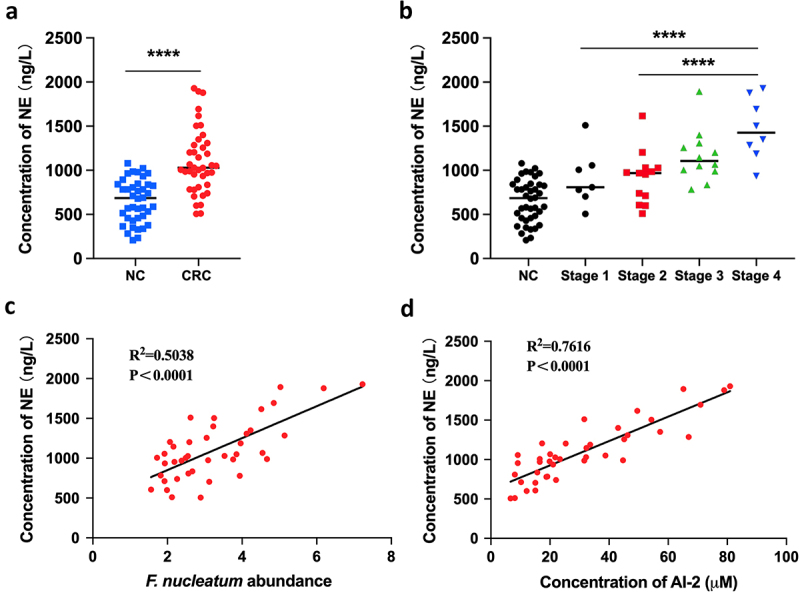
NE increased with the progression of *F. nucleatum* related CRC (a) the concentration of NE was significantly higher in CRC tissues (*n* = 40) compared with NC tissues (*n* = 40). (b) the concentration of NE in tumours increased along with the progression of CRC. (c, d) Pearson’s correlation analysis showed positive correlation between NE concentration and *F. nucleatum* abundance (c) or AI-2 concentration (d) in CRC tissues.

We determined the concentrations of NE in both tumour and adjacent normal tissues using ELISA. The concentration of NE was significantly higher in the tumours (*n* = 40) than in the adjacent normal tissues (*n* = 40, *p* < 0.0001) (Figure 2a).

****P < 0.0001, **P < 0.01. CRC, colorectal cancer; NC, adjacent normal control; NE, norepinephrine; *F. nucleatum*, *Fusobacterium nucleatum*; AI-2, auto-inducer-2.

Notably, the concentration of NE increased with CRC progression (Figure 2b). Additionally, we found a positive correlation between NE concentration and *F. nucleatum* abundance (Pearson’s correlation, *r* = 0.5038; *p* < 0.0001), as well as between NE concentration and AI-2 concentration (Pearson’s correlation, *r* = 0.7616; *p* < 0.0001) (Figure 2c,d). These results suggest that host-derived NE may be associated with the progression of *F. nucleatum* related CRC, possibly involving bacterial AI-2 signalling.

### NE promoted the virulence and proliferation of *F. nucleatum* with upregulation of QS system

**Figure 3.**
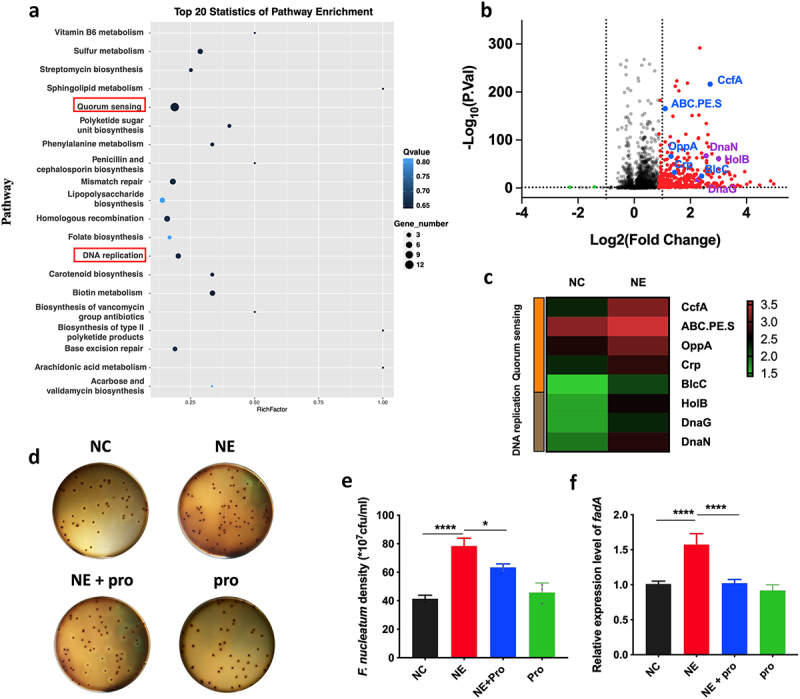
NE promoted the virulence and proliferation of *F. nucleatum* with upregulation of QS system (a) *F. nucleatum* was treated with NE for 24 hours followed by subjection to RNA-Seq. KEGG analysis of pathway enrichment showed the induction of QS after NE treatment. (b) volcano plot of the transcriptome in NE group versus NC group. QS associated genes (*ccfA*, *ABC.PE.S*, *oppA*, *crp*, *blcC*) and DNA replication associated genes (*holB*, *dnaG*, *dnaN*) were labelled. (c) Heatmap of differentially regulated genes associated with QS and DNA replication. (d) Representative images of the colony formation of *F. nucleatum* subjected to NE and propranolol treatments. (e) statistics of *F. nucleatum* density based on the colonies in (d). (f) qRT-PCR data showed that *fadA* expression was increased significantly with treatment of NE, and decreased by propranolol. All experiments were repeated three times, and one-way analysis of variance was used.

To study the role of NE in *F. nucleatum* activity, we analysed the gene expression profile of *F. nucleatum* after NE treatment for 24 h. NE treatment induced a QS system that utilized autoinducers (AIs) (Figure 3a). The QS-associated genes *ccfA*, *ABC.PE.S*, and, to a lesser extent, *oppA*, *blcC*, and *crp* were among the upregulated genes in the volcano plot and heatmap (Figure 3b,c), which are consistent with previous studies [44–47]. Moreover, DNA replication was observed in the transcriptomic analysis, which may suggest proliferation of *F. nucleatum* after NE treatment (Figure 3a).

*P < 0.05, **P < 0.01, ***P < 0.001. *F. nucleatum*, *Fusobacterium nucleatum*; QS, quorum sensing; NC, Negative control; NE, norepinephrine; pro, propranolol, NE inhibitor.

Therefore, we tested whether NE affected the growth and virulence of *F. nucleatum*. Using a colony formation assay, we found that NE promoted *F. nucleatum* proliferation (Figure 3d,e), whereas propranolol, an NE inhibitor, reversed the growth rate (*p* < 0.05) (Figure 3d,e). *FadA* of *F. nucleatum*, a virulence factor that regulates cell adhesion and invasive properties, was upregulated in response to NE treatment (Fig. S2), whereas its inhibitor decreased *fadA* expression (*p* < 0.05) (Figure 3f). These findings suggest that host NE may enhance the growth and virulence of *F. nucleatum* possibly through bacterial QS signalling.

### AI-2 is essential for NE enhanced virulence and proliferation of *F. nucleatum*

**Figure 4.**
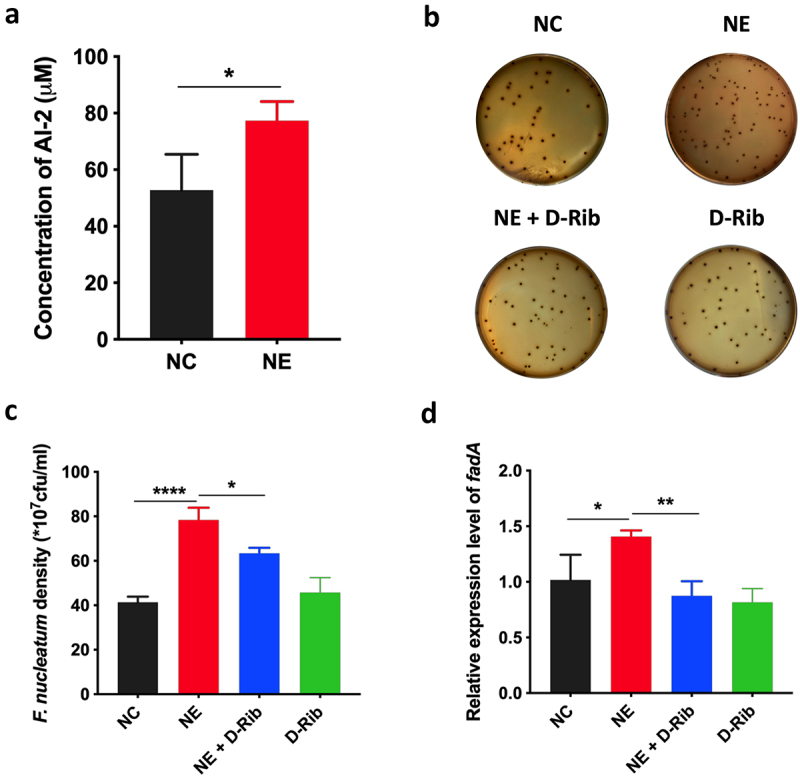
AI-2 is essential for NE enhanced virulence and proliferation of *F. nucleatum (a) statistics of AI-2 concentration in the supernatant of F. nucleatum culture induced by NE. (b) Representative images illustrating the colony formation of F. nucleatum subjected to NE treatments with and without AI-2 inhibitor. (c) statistics of F. nucleatum density based on the colonies in (b). (d) qRT-PCR data showed that D-Rib significantly inhibited the FadA expression of F. nucleatum induced by NE. All experiments were repeated three times, and paired t test was used*.

In addition, the level of AI-2 was significantly increased by NE (*p* < 0.05) (Figure 4a). Next, we treated *F. nucleatum* with D-Rib, an AI-2 competitive inhibitor, in combination with NE. The results showed that D-Rib inhibited the growth of *F. nucleatum* induced by NE (*p* < 0.05) (Figure 4b,c). Moreover, D-Rib significantly inhibited *fadA* expression of *F. nucleatum* induced by NE in F. nucleatum (*p* < 0.05) (Figure 4d). These results suggest that AI-2 signalling is required for the growth and virulence of *F. nucleatum* induced by NE.

*P < 0.05, **P < 0.01, ***P < 0.001. *F. nucleatum*, *Fusobacterium nucleatum*; NC, Negative control; NE, norepinephrine; D-Rib, D-Ribose; AI-2 competitive inhibitor.

### Stress accelerated the progression of colorectal tumor in Apc^Min/+^ mice infected with *F. nucleatum*

**Figure 5.**
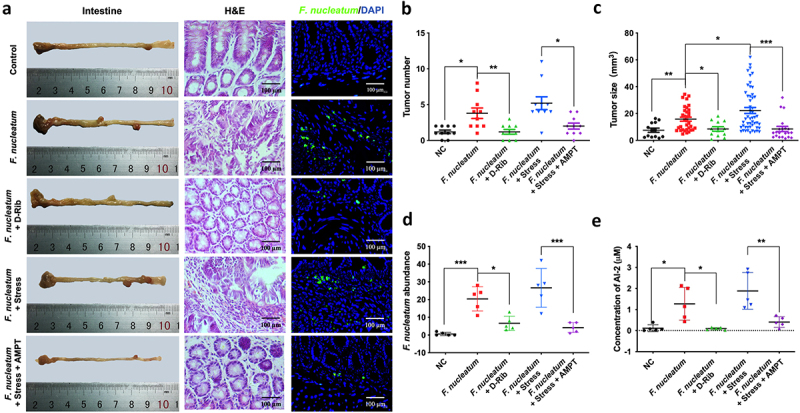
Stress promoted the progression of colorectal tumour in Apc^Min/+^ mice fed with *F. nucleatum (a) Representative images showing the tumor formation and F. nucleatum invasion in tumours. (b, c) quantification of tumour number and size in each group. D-Rib significantly decreased the tumour number and size in mice fed with F. nucleatum. the tumour size in F. nucleatum + stress group was significantly higher than in F. nucleatum group. Both the tumour number and size were decreased significantly by catecholamine inhibitor AMPT. (d) qRT-PCR analysis highlighting F. nucleatum burden in tumours. AMPT significantly decreased the abundance of F. nucleatum induced by host stress. (e) F. nucleatum administration significantly increased AI-2 level in tumours, which was inhibited by D-Rib or AMPT*.

Our previous studies have demonstrated that *F. nucleatum* administration can potentiate colorectal tumorigenesis in mice [48], and that AI-2 concentration was increased in both colorectal tissue and stool of CRC patients [20]. To investigate the role of AI-2 in *F. nucleatum* related CRC *in vivo*, we treated *F. nucleatum* fed Apc^Min/+^ mice with D-Rib, an AI-2 inhibitor. The tumour number and size in the intestine were lower in D-Rib-treated mice than in *F. nucleatum* group (*p* < 0.01, *p* < 0.05, respectively), suggesting that AI-2 signalling is required to enhance *F. nucleatum* induced colorectal tumours *in vivo* (Figure 5a,b,).

***P < 0.001, **P < 0.01. *P < 0.05. *F. nucleatum*, *Fusobacterium nucleatum*; D-Rib, D-Ribose; AMPT, catecholamine inhibitor; NC, Negative control.

To investigate the role of NE in *F. nucleatum* related colorectal tumours *in vivo*, we used a mouse model in which NE production was induced by subjecting the mice to chronic stress. The concentration of NE significantly increased in the tumours of mice subjected to stress (*p* < 0.05), which was counteracted by AMPT treatment (*p* < 0.01) (Fig. S3). Based on *F. nucleatum* administration, chronic stress accelerated intestinal tumour formation, with increased tumour number (although not significant) and size (*p* < 0.01) (Figure 5a,b,). Moreover, both the tumour number and size were significantly decreased by the NE inhibitor AMPT (*p* < 0.05) (Figure 5a,b). Additionally, AMPT significantly decreased the abundance of *F. nucleatum* induced by host stress (*p* < 0.001) (Figure 5d). In this mouse model, we also evaluated the concentration of AI-2, showing that *F. nucleatum* fed mice showed higher AI-2 level, which was rescued by D-Rib or AMPT (Figure 5e).

## Discussion

Previous studies have reported that *F. nucleatum* is enriched in tumour tissues and stools of CRC patients and is positively correlated with tumour stage [3]. However, as an opportunistic pathogen, *F. nucleatum* also exists in the healthy gut, raising the question of how *F. nucleatum* pathogenicity in CRC is regulated by host factors. In the present study, we found that the stress hormone NE can support the proliferation and virulence of *F. nucleatum in vitro* and mainly drive an increase in tumour size in APC^Min/+^ mice fed *F. nucleatum*, which was counteracted by the catecholamine inhibitor AMPT *in vivo* and *in vitro*. These results suggest that the host stress hormone NE promotes the progression of *F. nucleatum* related CRC.

It has been shown that *F. nucleatum* adhere to and invade epithelial cells via virulence factors, including *fadA*, Fusobacterium autotransporter protein 2 (Fap2), and Fusobacterial outer membrane protein A (FomA) [9,49–51]. Here, we found that NE promoted the growth of *F. nucleatum in vitro* and induced *fadA* expression. These effects were inhibited by an AI-2 inhibitor, suggesting that the pathogenicity of *F. nucleatum* is regulated by QS signalling.

Although it has been reported that stress promotes CRC progression [52], the underlying mechanism remains to be elucidated, especially in *F. nucleatum* related CRCs. Our results revealed a positive correlation between NE concentrations and AI-2 levels in human CRC tissues. We further analysed the gene expression profile of *F. nucleatum* after NE treatment and found that QS-associated genes, such as *ccfA* and *ABC. PE. S*, were upregulated. These interesting findings indicate, for the first time, that host stress may promote the progression of *F. nucleatum* related CRC through NE/AI-2 signalling.

To our knowledge, this is the first study to address the mechanism of *F. nucleatum* related CRC with a focus on the brain-gut axis and the inter-kingdom signalling communication between the host neuroendocrine system and bacteria. Our results suggest a new strategy for the management of *F. nucleatum* related CRC by targeting inter-kingdom signalling between host stress hormones and the bacterial QS system. This strategy may also benefit other diseases associated with brain-gut axis dysfunction or gut microbiota dysbiosis, such as inflammatory bowel disease (IBD) and irritable bowel syndrome (IBS).

## Supplementary Material

Fig S4.tiff

Fig S1.tif

Fig S2.tiff

Supplementary table S1.docx

Fig S3.tif

## Data Availability

The authors confirm that the data supporting the findings of this study are available in the article and its supplementary materials.

## References

[1] Larroya-García A, Navas-Carrillo D, Orenes-Piñero E. Impact of gut microbiota on neurological diseases: diet composition and novel treatments. Crit Rev Food Sci Nutr. 2018;59(19):3102–11. doi: 10.1080/10408398.2018.1484340 29870270

[2] Siegel RL, Miller KD, Jemal A. Cancer statistics, 2019. Ca A Cancer J Clinicians. 2019;69(1):7–34. doi: 10.3322/caac.21551 30620402

[3] Wu J, Li Q, Fu X. Fusobacterium nucleatum contributes to the carcinogenesis of colorectal cancer by inducing inflammation and suppressing Host immunity. Transl Oncol. 2019;12(6):846–851. doi: 10.1016/j.tranon.2019.03.003 30986689 PMC6462820

[4] Chen T, Li Q, Zhang X, et al. TOX expression decreases with progression of colorectal cancers and is associated with CD4 T-cell density and Fusobacterium nucleatum infection. Hum Pathol. 2018;79:93–101. doi: 10.1016/j.humpath.2018.05.008 29792893

[5] Chen Y, Peng Y, Yu J, et al. Invasive Fusobacterium nucleatum activates beta-catenin signaling in colorectal cancer via a TLR4/P-PAK1 cascade. Oncotarget. 2017;8(19):31802–31814. doi: 10.18632/oncotarget.15992 28423670 PMC5458249

[6] Kostic A, Chun E, Robertson L, et al. Fusobacterium nucleatum potentiates intestinal tumorigenesis and modulates the tumor-immune microenvironment. Cell Host Microbe. 2013;14(2):207–215. doi: 10.1016/j.chom.2013.07.007 23954159 PMC3772512

[7] Yang Y, Weng W, Peng J, et al. Fusobacterium nucleatum increases proliferation of colorectal cancer cells and tumor development in mice by activating toll-like receptor 4 signaling to nuclear Factor−κB, and up-regulating expression of MicroRNA-21. Gastroenterology. 2017;152(4):851–866.e24. doi: 10.1053/j.gastro.2016.11.018 27876571 PMC5555435

[8] Dadashi M, Hajikhani B, Faghihloo E, et al. Proliferative effect of FadA recombinant protein from Fusobacterium nucleatum on SW480 colorectal cancer cell line. Infect Disord Drug Targets. 2021;21(4):623–628. doi: 10.2174/1871526520666200720113004 32691717

[9] Rubinstein MR, Wang X, Liu W, et al. Fusobacterium nucleatum promotes colorectal carcinogenesis by modulating E-cadherin/β-catenin signaling via its FadA adhesin. Cell Host Microbe. 2013;14(2):195–206. doi: 10.1016/j.chom.2013.07.012 23954158 PMC3770529

[10] Rubinstein MR, Baik JE, Lagana SM, et al. Fusobacterium nucleatum promotes colorectal cancer by inducing Wnt/β-catenin modulator annexin A1. EMBO Rep. 2019;20(4):e47638. doi: 10.15252/embr.201847638 30833345 PMC6446206

[11] Yu T, Guo F, Yu Y, et al. Fusobacterium nucleatum promotes chemoresistance to colorectal cancer by modulating autophagy. Cell. 2017;170(3):548–563.e16. doi: 10.1016/j.cell.2017.07.008 28753429 PMC5767127

[12] Bashir A, Miskeen AY, Bhat A, et al. Fusobacterium nucleatum: an emerging bug in colorectal tumorigenesis. Eur J Cancer Prev. 2015;24(5):373–385. doi: 10.1097/CEJ.0000000000000116 25569450

[13] Thompson JA, Oliveira R, Djukovic A, et al. Manipulation of the Quorum Sensing Signal AI-2 affects the Antibiotic-Treated Gut Microbiota. Cell Rep. 2015;10(11):1861–1871. doi: 10.1016/j.celrep.2015.02.049 25801025

[14] Fuqua WC, Winans SC, Greenberg EP. Quorum sensing in bacteria: the LuxR-LuxI family of cell density-responsive transcriptional regulators. J Bacteriol. 1994;176(2):269–275. doi: 10.1128/jb.176.2.269-275.1994 8288518 PMC205046

[15] Rettner RE, Saier MH, Jr. The autoinducer-2 exporter superfamily. J Microb Physiol. 2010;18(4):195–205. doi: 10.1159/000316420 PMC3004238 20559013

[16] Wang Y, Wang Y, Sun L, et al. The LuxS/AI-2 system of streptococcus suis. Appl Microbiol Biotechnol. 2018;102(17):7231–7238. doi: 10.1007/s00253-018-9170-7 29938319

[17] Li Q, Ren Y, Fu X. Inter-kingdom signaling between gut microbiota and their host. Cell Mol Life Sci. 2019;76(12):2383–2389. doi: 10.1007/s00018-019-03076-7 30911771 PMC11105296

[18] Jang Y, Choi Y-J, Lee S-H, et al. Autoinducer 2 of Fusobacterium nucleatum as a target molecule to inhibit biofilm formation of periodontopathogens. Arch Oral Biol. 2013;58(1):17–27. doi: 10.1016/j.archoralbio.2012.04.016 22633049

[19] Wu J, Wang Y, Jiang Z. Fusobacterium nucleatumImmune induction identified by TMT proteomics analysis in autoinducer-2 treated macrophages. Expert Rev Proteomics. 2020;17(2):175–185. doi: 10.1080/14789450.2020.1738223 32125181

[20] Li Q, Peng W, Wu J, et al. Autoinducer-2 of gut microbiota, a potential novel marker for human colorectal cancer, is associated with the activation of TNFSF9 signaling in macrophages. Oncoimmunology. 2019;8(10):e1626192. doi: 10.1080/2162402X.2019.1626192 31646072 PMC6791418

[21] Zeng X, Yue H, Zhang L, et al. Gut microbiota-derived autoinducer-2 regulates lung inflammation through the gut-lung axis. Int Immunopharmacol. 2023;124:110971. doi: 10.1016/j.intimp.2023.110971 37748222

[22] Dicks LMT. How does quorum sensing of intestinal bacteria affect our health and mental status? Microorganisms. 2022;10(10):1969. doi: 10.3390/microorganisms10101969 36296244 PMC9611604

[23] Martin AM, Sun EW, Keating DJ. Mechanisms controlling hormone secretion in human gut and its relevance to metabolism. J Endocrinol. 2020;244(1):R1–R15. doi: 10.1530/JOE-19-0399 PMC6892457 31751295

[24] Rehfeld JF. Gastrointestinal hormones and their targets. Adv Exp Med Biol. 2014;817:157–175. 24997033 10.1007/978-1-4939-0897-4_7

[25] Asano Y, Hiramoto T, Nishino R, et al. Critical role of gut microbiota in the production of biologically active, free catecholamines in the gut lumen of mice. Am J Physiol Gastrointest Liver Physiol. 2012;303(11):G1288–G1295. doi: 10.1152/ajpgi.00341.2012 23064760

[26] Moreira CG, Russell, R., Mishra, A.A., et al. Bacterial adrenergic sensors regulate virulence of enteric pathogens in the gut. MBio. 2016;7(3):e00826–16. 27273829 10.1128/mBio.00826-16 PMC4959670

[27] Boukerb AM, Cambronel M, Rodrigues S, et al. Inter-kingdom signaling of stress hormones: sensing, transport and modulation of bacterial physiology. Front Microbiol. 2021;12:690942. doi: 10.3389/fmicb.2021.690942 34690943 PMC8526972

[28] Mittal R, Debs LH, Patel AP, et al. Neurotransmitters: the Critical Modulators Regulating Gut–brain axis. J Cell Physiol. 2017;232(9):2359–2372. doi: 10.1002/jcp.25518 27512962 PMC5772764

[29] Roberts A, Matthews JB, Socransky SS, et al. Stress and the periodontal diseases: effects of catecholamines on the growth of periodontal bacteria in vitro. Oral Microbiol Immunol. 2002;17(5):296–303. doi: 10.1034/j.1399-302X.2002.170506.x 12354211

[30] Kinney KS, Austin CE, Morton DS, et al. Norepinephrine as a growth stimulating factor in bacteria—mechanistic studies. Life Sci. 2000;67(25):3075–3085. doi: 10.1016/S0024-3205(00)00891-2 11125844

[31] Lyte M, Frank CD, Green BT. Production of an autoinducer of growth by norepinephrine cultured Escherichia coli O157: H7. FEMS Microbiol Lett. 1996;139(2–3):155–159. doi: 10.1111/j.1574-6968.1996.tb08196.x 8674983

[32] Raut N, Joel S, Pasini P, et al. Bacterial autoinducer-2 detection via an engineered quorum sensing protein. Anal Chem. 2015;87(5):2608. doi: 10.1021/ac504172f 25654248

[33] Chen Y, Chen Y, Zhang J, et al. Fusobacterium nucleatum promotes metastasis in colorectal cancer by activating autophagy signaling via the Upregulation of CARD3 Expression. Theranostics. 2020;10(1):323–339. doi: 10.7150/thno.38870 31903123 PMC6929621

[34] Parhi L, Alon-Maimon T, Sol A, et al. Breast cancer colonization by Fusobacterium nucleatum accelerates tumor growth and metastatic progression. Nat Commun. 2020;11(1):3259. doi: 10.1038/s41467-020-16967-2 32591509 PMC7320135

[35] Schmittgen T, Livak K. Analyzing real-time PCR data by the comparative C(T) method. Nat Protoc. 2008;3(6):1101–1108. doi: 10.1038/nprot.2008.73 18546601

[36] Yu YN, Yu T-C, Zhao H-J, et al. Berberine may rescue Fusobacterium nucleatum-induced colorectal tumorigenesis by modulating the tumor microenvironment. Oncotarget. 2015;6(31):32013–32026. doi: 10.18632/oncotarget.5166 26397137 PMC4741656

[37] Bassler BL, Greenberg EP, Stevens AM. Cross-species induction of luminescence in the quorum-sensing bacterium vibrio harveyi. J Bacteriol. 1997;179(12):4043–4045. doi: 10.1128/jb.179.12.4043-4045.1997 9190823 PMC179216

[38] Stephens K, Pozo M, Tsao C-Y, et al. Bacterial co-culture with cell signaling translator and growth controller modules for autonomously regulated culture composition. Nat Commun. 2019;10(1):4129. doi: 10.1038/s41467-019-12027-6 31511505 PMC6739400

[39] Song J, Qin Q, Li T, et al. Impact of carbohydrates on autoinducer-2 secretion of Bifidobacterium longum subsp. longum BBMN68. Lett Appl Microbiol. 2018;66(4):340–346. doi: 10.1111/lam.12854 29356014

[40] James D, Shao H, Lamont RJ, et al. The Actinobacillus actinomycetemcomitans ribose binding protein RbsB interacts with cognate and heterologous autoinducer 2 signals. Infect Immun. 2006;74(7):4021–4029. doi: 10.1128/IAI.01741-05 16790775 PMC1489740

[41] Liu J, Deng G-H, Zhang J, et al. The effect of chronic stress on anti-angiogenesis of sunitinib in colorectal cancer models. Psychoneuroendocrinology. 2015;52:130–142. doi: 10.1016/j.psyneuen.2014.11.008 25437118

[42] Perego M, Tyurin VA, Tyurina YY, et al. Reactivation of dormant tumor cells by modified lipids derived from stress-activated neutrophils. Sci Transl Med. 2020;12(572):eabb5817. doi: 10.1126/scitranslmed.abb5817 33268511 PMC8085740

[43] Weir G, Ramage LE, Akyol M, et al. Substantial metabolic activity of human brown adipose tissue during warm conditions and cold-induced lipolysis of local triglycerides. Cell Metab. 2018;27(6):1348–1355.e4. doi: 10.1016/j.cmet.2018.04.020 29805098 PMC5988566

[44] Khan SR, Farrand SK. The BlcC (AttM) lactonase of agrobacterium tumefaciens does not quench the quorum-sensing system that regulates Ti plasmid conjugative transfer. J Bacteriol. 2009;191(4):1320–1329. doi: 10.1128/JB.01304-08 19011037 PMC2632005

[45] Kurnia D, Rachmawati P, Satari MH. Antibacterial of dibenzo-p-dioxi-2,8-dicarboxylic acid against pathogenic oral bacteria E. faecalis ATCC 29212 peptide pheromones: quorum sensing of in vitro and in silico study. Drug Des Devel Ther. 2020;14:3079–3086. 10.2147/DDDT.S255270 PMC7398749 32801646

[46] Laganenka L, Sander T, Lagonenko A, et al. Quorum sensing and metabolic state of the Host Control Lysogeny-Lysis switch of bacteriophage T1. MBio. 2019;10(5):e01884–19. doi: 10.1128/mBio.01884-19 31506310 PMC6737242

[47] Yang M, Meng F, Gu W, et al. Influence of polysaccharides from polygonatum kingianum on Short-Chain Fatty Acid Production and quorum sensing in lactobacillus faecis. Front Microbiol. 2021;12:758870. doi: 10.3389/fmicb.2021.758870 34867887 PMC8635744

[48] Wu Y, Wu J, Chen T, et al. Fusobacterium nucleatum potentiates intestinal tumorigenesis in mice via a toll-like receptor 4/p21-activated kinase 1 cascade. Dig Dis Sci. 2018;63(5):1210–1218. doi: 10.1007/s10620-018-4999-2 29508166

[49] Abed J, Emgård JM, Zamir G, et al. Fap2 mediates Fusobacterium nucleatum colorectal adenocarcinoma enrichment by binding to tumor-expressed gal-GalNAc. Cell Host Microbe. 2016;20(2):215–225. doi: 10.1016/j.chom.2016.07.006 27512904 PMC5465824

[50] Gur C, Ibrahim Y, Isaacson B, et al. Binding of the Fap2 protein of Fusobacterium nucleatum to human inhibitory receptor TIGIT protects tumors from immune cell attack. Immunity. 2015;42(2):344–355. doi: 10.1016/j.immuni.2015.01.010 25680274 PMC4361732

[51] Nakagaki H, Sekine S, Terao Y, et al. Fusobacterium nucleatum envelope protein FomA is immunogenic and binds to the salivary statherin-derived peptide. Infect Immun. 2010;78(3):1185–1192. doi: 10.1128/IAI.01224-09 20008529 PMC2825909

[52] Bandapalli OR, Xu J, Liang F, et al. Effect of chronic psychological stress on liver metastasis of colon cancer in mice. PLOS ONE. 2015;10(10):e0139978. doi: 10.1371/journal.pone.0139978 26444281 PMC4596521

