# Supplementary figures and images for: Norepinephrine may promote the progression of Fusobacterium nucleatum related colorectal cancer via quorum sensing signalling
Source: Virulence. 2024 May 9;15(1):2350904. doi: 10.1080/21505594.2024.2350904 (PMC11085999; doi:10.1080/21505594.2024.2350904)

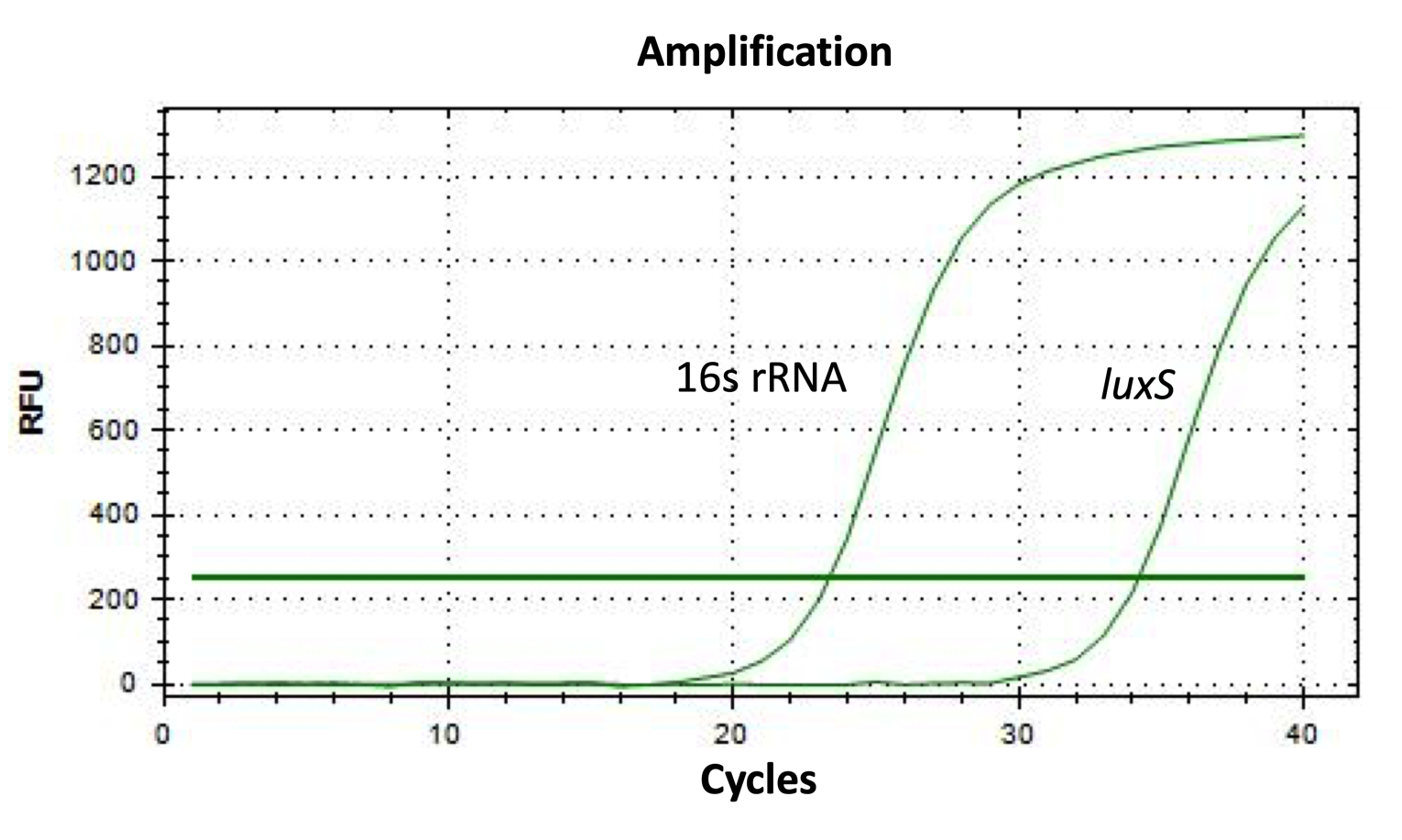

Supplement: Fig S4.tiff [file KVIR_A_2350904_SM5524.tiff]

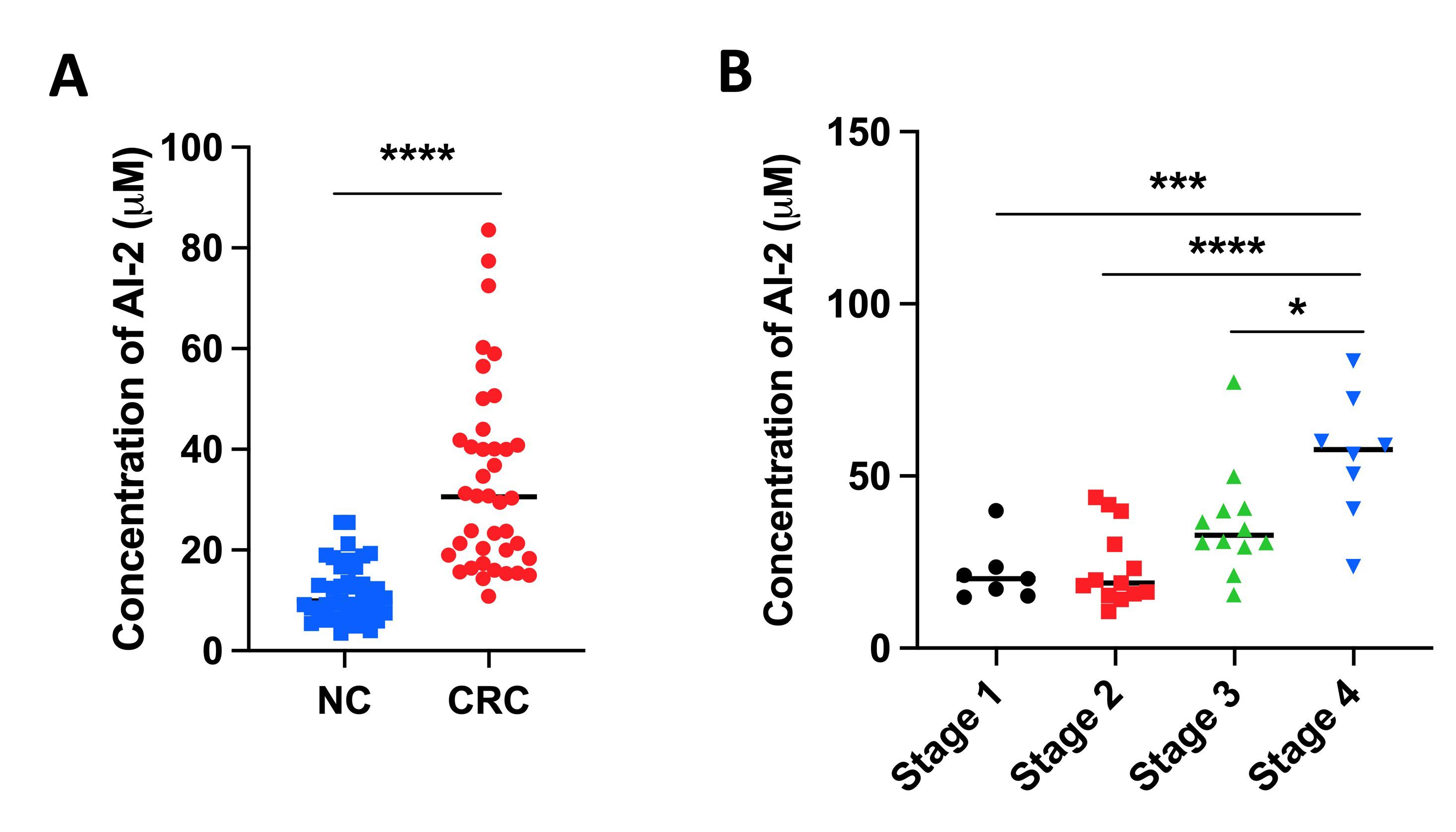

Supplement: Fig S1.tif [file KVIR_A_2350904_SM5523.tif]

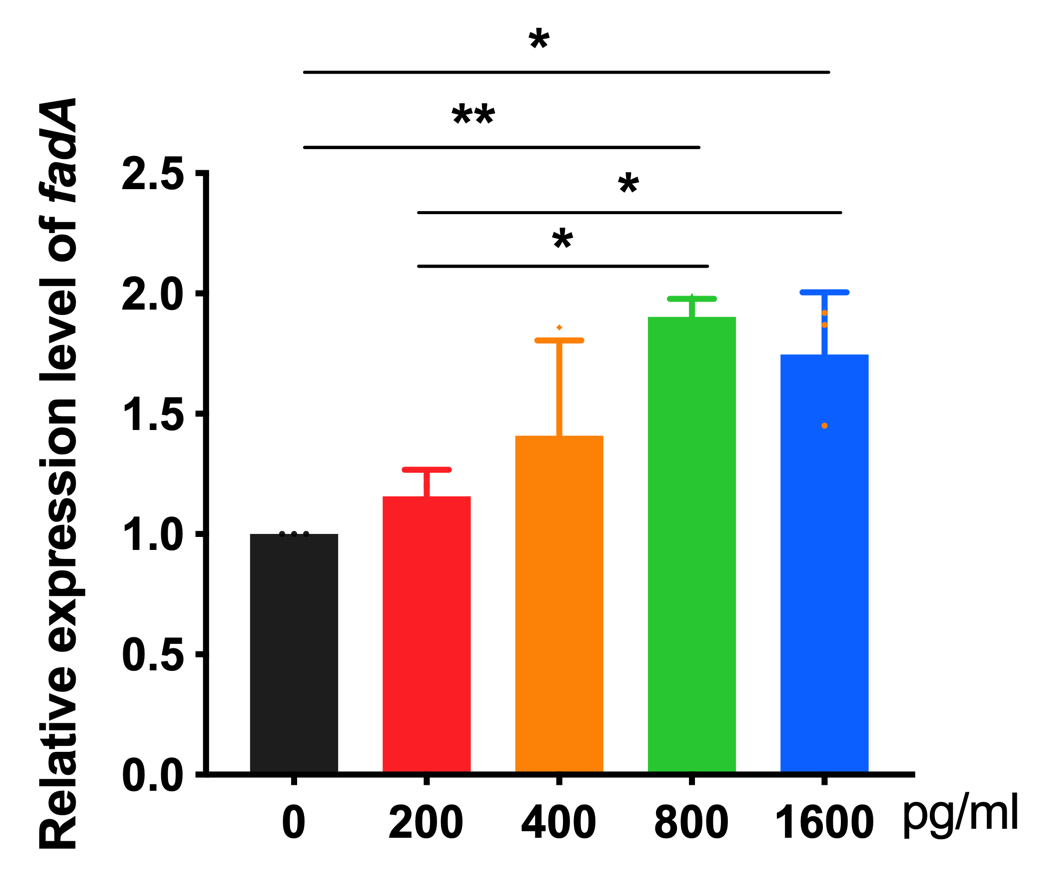

Supplement: Fig S2.tiff [file KVIR_A_2350904_SM5522.tiff]

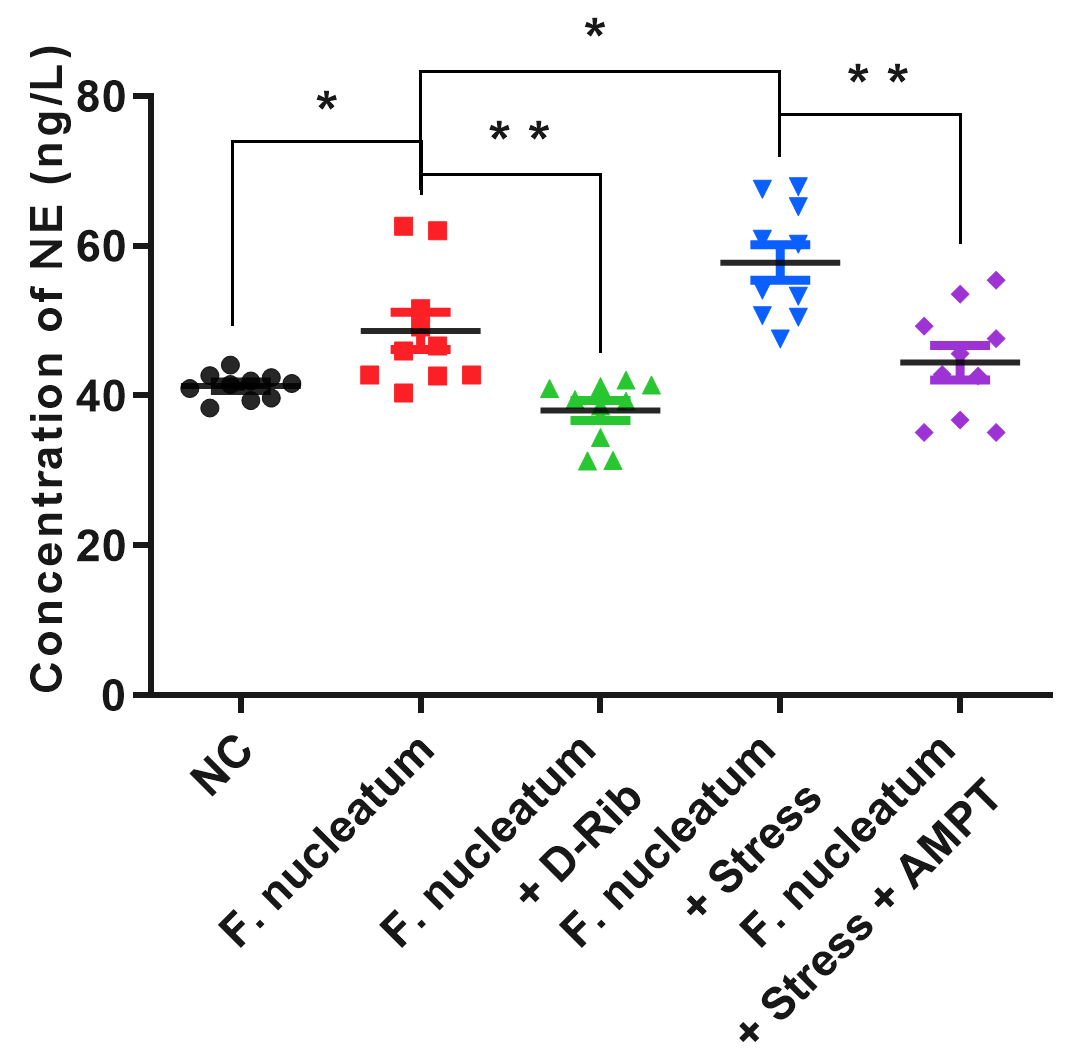

Supplement: Fig S3.tif [file KVIR_A_2350904_SM5520.tif]
