# Supplementary material for: Norepinephrine may promote the progression of Fusobacterium nucleatum related colorectal cancer via quorum sensing signalling
Source: Virulence. 2024 May 9;15(1):2350904. doi: 10.1080/21505594.2024.2350904 (PMC11085999; doi:10.1080/21505594.2024.2350904)
Supplement: Supplementary table S1.docx [file KVIR_A_2350904_SM5521.docx]

**Supplementary Table S1. The basic characteristics of patients.**

|  | **CRC patients**  **(n=40)** | **Healthy volunteers**  **(n=38)** | ***P* value** |
| --- | --- | --- | --- |
| Gender |  |  |  |
| Female (n) | 24 | 23 | 0.8213 |
| Male (n) | 16 | 15 |  |
| Average age (year) | 59.23±10.30 | 58.08±8.514 |  |
| Location |  |  |  |
| Right location | 9 | NA |  |
| Left location | 31 | NA |  |
| TNM staging |  |  |  |
| I | 4 | NA |  |
| II | 15 | NA |  |
| III | 16 | NA |  |
| IV | 5 | NA |  |
| Differentiation |  |  |  |
| WD | 9 | NA |  |
| MPD | 31 | NA |  |
| Metastasis |  |  |  |
| Non-LNM | 19 | NA |  |
| LNM | 21 | NA |  |
| Smoking |  |  |  |
| Absence | 28 | 24 | 0.3584 |
| Presence | 12 | 16 |  |
| Drinking |  |  |  |
| Never | 12 | 15 | 0.8778 |
| Occasionally | 22 | 20 |  |
| Frequently | 6 | 5 |  |

AD, adenoma; CRC, colorectal cancer; LNM, lymph node metastasis; MPD, moderately and poorly differentiated; NA, not applicable; Non-LNM, non-lymph node metastasis; WD, well differentiated.
